# The effects of implementing synoptic pathology reporting in cancer diagnosis: a systematic review

**DOI:** 10.1007/s00428-016-1935-8

**Published:** 2016-04-21

**Authors:** Caro E. Sluijter, Luc R. C. W. van Lonkhuijzen, Henk-Jan van Slooten, Iris D. Nagtegaal, Lucy I. H. Overbeek

**Affiliations:** Department of Pathology, Radboud University Medical Centre, Huispost 824, P.O. Box 9101, 6500 HB Nijmegen, The Netherlands; Foundation PALGA (The Nationwide Network and Registry of Histo- and Cytopathology in the Netherlands), Houten, The Netherlands; Centre for Gynaecological Oncology, Academic Medical Centre, Amsterdam, The Netherlands; Symbiant Pathology Expert Centre, Alkmaar, The Netherlands

**Keywords:** Pathology, Synoptic reporting, Narrative reporting, Checklist, Template, Proforma, Guideline, Completeness, Quality, Colorectal carcinoma, Breast cancer

## Abstract

Pathology reporting is evolving from a traditional narrative report to a more structured synoptic report. Narrative reporting can cause misinterpretation due to lack of information and structure. In this systematic review, we evaluate the impact of synoptic reporting on completeness of pathology reports and quality of pathology evaluation for solid tumours. Pubmed, Embase and Cochrane databases were systematically searched to identify studies describing the effect of synoptic reporting implementation on completeness of reporting and quality of pathology evaluation of solid malignant tumours. Thirty-three studies met the inclusion criteria. All studies, except one, reported an increased overall completeness of pathology reports after introduction of synoptic reporting (SR). Most frequently studied cancers were breast (*n* = 9) and colorectal cancer (*n* = 16). For breast cancer, narrative reports adequately described ‘tumour type’ and ‘nodal status’. Synoptic reporting resulted in improved description of ‘resection margins’, ‘DCIS size’, ‘location’ and ‘presence of calcifications’. For colorectal cancer, narrative reports adequately reported ‘tumour type’, ‘invasion depth’, ‘lymph node counts’ and ‘nodal status’. Synoptic reporting resulted in increased reporting of ‘circumferential margin’, ‘resection margin’, ‘perineural invasion’ and ‘lymphovascular invasion’. In addition, increased numbers of reported lymph nodes were found in synoptic reports. Narrative reports of other cancer types described the traditional parameters adequately, whereas for ‘resection margins’ and ‘(lympho)vascular/perineural invasion’, implementation of synoptic reporting was necessary. Synoptic reporting results in improved reporting of clinical relevant data. Demonstration of clinical impact of this improved method of pathology reporting is required for successful introduction and implementation in daily pathology practice.

**Electronic supplementary material:**

The online version of this article (doi:10.1007/s00428-016-1935-8) contains supplementary material, which is available to authorized users.

## Introduction

The ever increasing complexity of cancer treatment requires a high-quality diagnostic process, in which anatomic pathology plays a central role. A complete and clear anatomic pathology report forms the basis for optimal treatment decisions [1]. Depending on cancer type, an increasing number of parameters need to be reported by pathologists [2–5].

The way anatomic pathology reports are constructed needs to adapt to the continuous increase in complexity of reported diagnostic data [6]. There is a spectrum in the way pathology results are reported. This spectrum is divided into six levels by Srigley et al. [6]. Traditionally, a report consists of the following three paragraphs: macroscopy, microscopy and conclusion all completed with free text and without any further guidelines. These traditional narrative pathology reports (NRs) are considered level 1 reporting. NRs are still the standard in most jurisdictions, even though they are prone to misinterpretation [7] and do not always contain all mandatory information [8–16]. Level three consists of a synoptic-like structured format. With this method, the pathologist follows a checklist per cancer type to ensure that all mandatory parameters are reported. The layout of this type of reporting can still be narrative. More recently, synoptic reporting (SR) has been introduced in pathology. With SR, an electronic reporting module is used with standardised reporting language, multiple-choice answering of mandatory pathology parameters and automated generation of the conclusion (such as TNM stage). Generating a diagnostic report using such a system is much more comparable to filling out a form in an internet browser than it is to narrative reporting using speech recognition software. The result is a well-structured overview of the mandatory parameters for the pathology report (level 6). All levels are described in detail by Srigley et al. [6].

SR has been implemented in several settings all over the world [17]. However, an overview of the effect of SR on the completeness of pathology reports and quality of pathology evaluation in cancer diagnosis is lacking. In the current review, we evaluated the impact of the introduction of SR. We hypothesised that the implementation of SR improved both the completeness of anatomic pathology reports (per parameter and overall) as well as the inherent quality of anatomic pathologic evaluation of cancer specimens.

## Materials and methods

To identify studies that described the effect of SR on completeness of reporting and quality of pathology evaluation of solid malignant tumours, a systematic literature search was performed.

### Literature search

A combination of search terms in Pubmed, Embase and Cochrane was used to perform the literature search. For the search, we included variations of the following terms: ‘synoptic’, ‘checklist’, ‘template’, ‘pathologic’, ‘histopathology’ and ‘report’. In addition, reference lists of selected papers were manually searched (Online resource 1 describes the search terms in detail). The literature search was performed on September 30, 2015.

Studies were included if studies investigated human subjects, pathology, solid tumours, SR and histology. Selection was first based on title and subsequently on abstract. Only original studies evaluating the effect of SR versus NR of solid malignant tumours were selected. (Conference) abstracts, case reports, editorials, letters and studies for which the full text was not available were excluded. Only studies describing quantitative outcomes of the comparison of SR with NR were included. Therefore, we excluded studies that only described a format of pathology reporting before implementation of SR that described the development of a SR module or the implementation strategy for SR. Two independent investigators (CS and LvL) reviewed each full text report for eligibility.

From each included article, data was extracted on country of study, year and period of study, study design, cancer type, level of reporting before and after the implementation of SR [6], origin of guideline on which the synoptic data parameters are based, outcome measures, results and authors’ conclusion. The format or level of SR as described by Srigley et al. [6] was determined to categorise the studies.

### Outcome measures

The outcome measures evaluated in this systematic review were completeness of the pathology reports and the quality of pathology evaluation. We used two definitions for completeness of pathology reports: (1) overall completeness, the proportion of pathology reports containing all mandatory pathology parameters in a given time frame, and (2) parameter-specific completeness, the proportion of pathology reports in which an individual parameter was present in a given time frame. Both definitions were applied to the selected studies.

Quality of pathology evaluation was defined as the proportion of pathology reports in which the informational content corresponds to established quality indicators, such as lymph node numbers, presence of extramural vascular invasion and resection margins.

### Data evaluation

The studies were categorised based on cancer type and the implemented level of SR (level 3 versus ≥level 4). To compare completeness, absolute numerical data in studies were converted into percentages. We included parameters that were reported in at least two independent studies. For readability, in the tables, we included only parameters that were reported in at least three independent studies. There is no established definition for sufficient reporting of a parameter. We considered a parameter sufficiently reported if the proportion of pathology reports containing the parameter was greater than 90 % in all the studies that studied the parameter, per cancer type. This percentage was based on definitions used in a number of other studies [18–22].

## Results

**Fig. 1:**
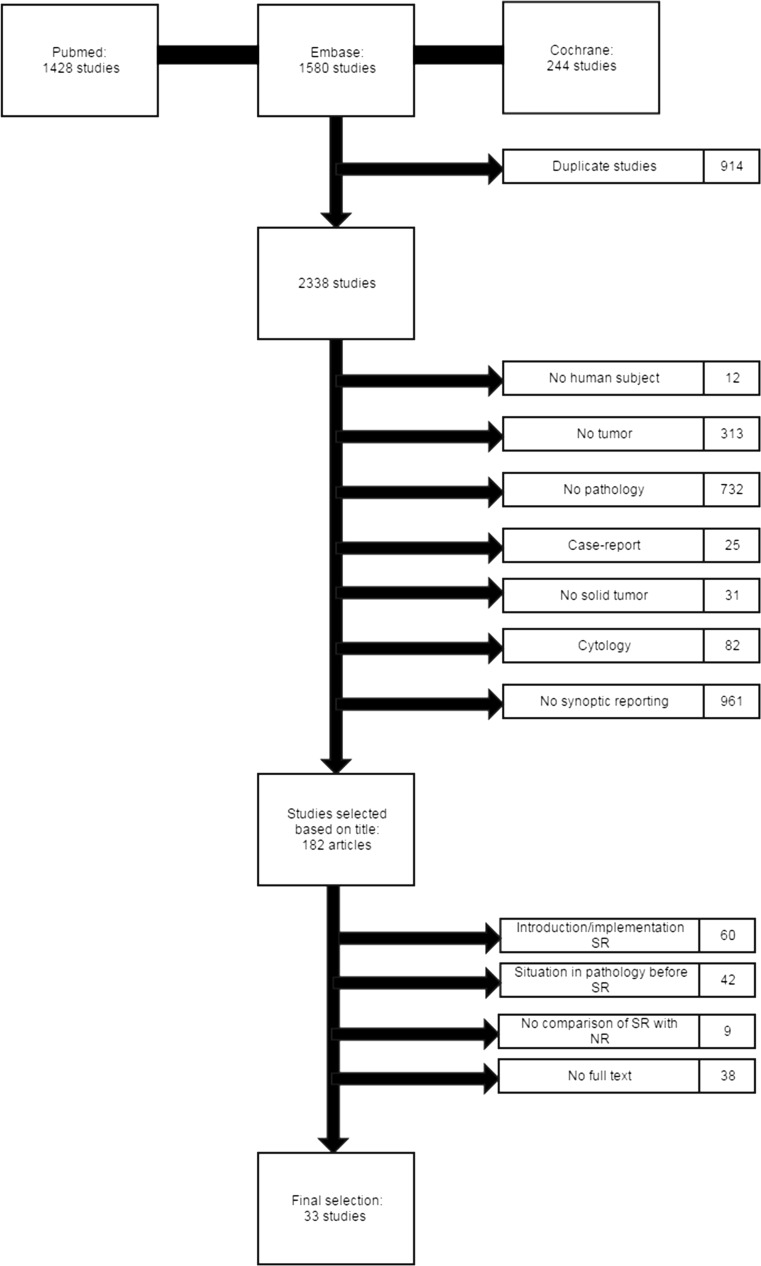
Flowchart of search strategy

A total of 3252 potentially relevant studies were retrieved by the database search. After removing duplicates, 2338 studies remained (Fig. 1). We excluded 2156 studies based on title, another 111 studies based on abstract or full text and 38 studies because the full-text article was not available. The remaining 33 studies were included for this review [6, 18–21, 23–52].

### Characteristics of studies

Table 1 summarises the characteristics of the 33 included studies. Twenty-three studies had a cross-sectional design and ten a case-control design. The studies originated from the following countries: the UK (*n* = 7), Australia (*n* = 6), Canada (*n* = 5), the USA (*n* = 4), Norway (*n* = 4), Germany (*n* = 2), the Czech Republic, Ireland, Italy, Sri Lanka and Sweden (all *n* = 1). Ten different types of cancer were covered in the studies. Most covered cancer types were colorectal (*n* = 16), breast (*n* = 9) and prostate cancers (*n* = 6). Twenty-three out of the 33 studies implemented a checklist format (level 3); the other ten studies implemented a higher SR level (≥level 4). Some studies described a two-step process of implementing SR level 4 or higher [6, 18, 23–25, 31, 35, 42, 47]. The SR modules were based on different guidelines, the College of American Pathologists (CAP; *n* = 12), the Royal College of Pathologists (RCP; *n* = 9) and other guidelines (*n* = 5). Some SR modules were based on expert opinion of a pathologist (*n* = 7).

**Table 1:** Characteristic of included studies

Article	Country	Cancer type	Number of subjects (*n*)	Study design (CC or CS)	Level synoptic reporting			Origin guidelines
					Before	Step 1	Step 2	
Appleton [23]	UK	Breast	40	CS	1	2	3	NHSBSP
Aumann [24]	Germany	Prostate	1049	CS	1	3	5	CAP
Aumann [25]	Germany	Lung	878	CS	1	3	5	CAP
Austin [19]	Australia	Breast	402	CS	1	3	–	ACN
Beattie [26]	Ireland	Colorectal	171	CC	1	3	–	RCP
Branston [28]	UK	Breast and colorectal	2042	CC	1	4	–	RCP
Buchwald [29]	Sweden	Colon	302	CC	1	3	–	Own
Casati [30]	Norway	Colorectal	1221	CC	1	1 and 5	–	RCP
Chan [18]	Canada	Colorectal	407	CS	1	3	4	CAP
Cross [31]	UK	Colorectal	272	CS	1	2	3	RCP
Gill [20]	Australia	Pancreatic	177	CC	1	3	–	CAP
Hammond [32]	USA	Breast	796	CS	1	3	–	Own
Hassel [33]	USA	Breast, prostate and melanoma	368	CC	1	4	–	CAP
Haugland [34]	Norway	Colorectal	650	CS	1	5	–	RCP
Haydu [35]	Australia	Melanoma	3784	CS	1	3	1 and 3	2008 Melanoma Guidelines
Idowu [36]	USA	Breast, colorectal and prostate	2125	CS	1	3	–	CAP
Ihnat [37]	Czech Republic	Colorectal	177	CC	1	3	–	CAP
Kahn [38]	Australia	Thyroid	448	CS	1	3	–	RCPA
Karim [39]	Australia	Melanoma	1692	CC	1	3	–	Own
Mathers [40]	UK	Breast	100	CC	1	3	–	RCP
McEvoy [41]	Australia	Breast	1649	CS	?	3	–	NHMRC
Messenger [42]	Canada	Rectal	498	CS	1	3	6	CAP
Porter, 2013 [43]	Canada	Rectal	197	CS	1	3	–	Own
Reid, 2000 [44]	UK	Uterine and cervix	349	CS	1	3	–	Own
Renshaw, 2014 [45]	USA	All synoptic pathology reports	6193	CS	1	2	3	CAP
Rigby, 2000 [46]	UK	Colorectal	98	CS	1	3	–	RCP
Siriwardana [21]	Sri Lanka	Colorectal	168	CS	1	3	–	RCP
Srigley [47]	Canada	Breast, lung, endometrium, colorectal, and prostate	7594	CS	1	3	6	CAP
Srigley [6]	Canada	Colorectal and prostate	All reports	CS	1	3	6	CAP
Ventura [48]	Italy	Prostate	70	CC	1	3	–	CAP
Westgaard [49]	Norway	Pancreatic	506	CS	1	3	–	Own
Westgaard [50]	Norway	Pancreatic	218	CS	1	3	–	Own
Woods [51]	UK	Colorectal	953	CS	1	3	–	RCP

*UK* United Kingdom, *USA* United States of America, *CC* case-control, *CS* cross sectoral, *NHSBSP* National Health Service Breast Screening Programme, *CAP* College of American Pathologists, *ACN* Australian Cancer Network, *RCP* Royal College of Pathologists, *RCPA* Royal College of Pathologists Australasia, *NHMRC* National Health and Medical Research Council

### Completeness of pathology reports

#### Overall completeness

**Fig. 2:**
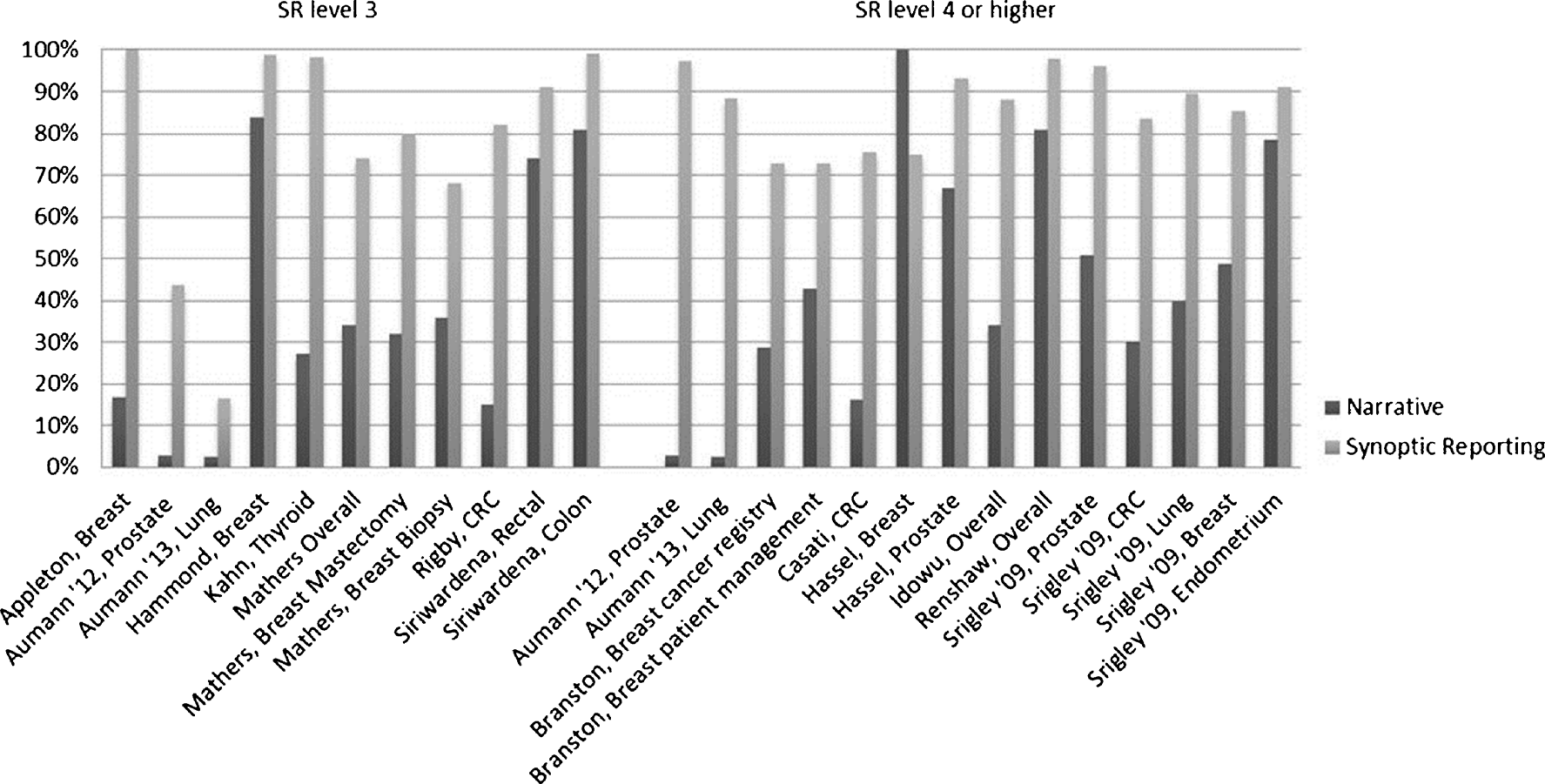
Impact of synoptic reporting on overall completeness of a pathology report. Fourteen studies [21, 23–25, 28, 30, 32, 33, 36, 38, 40, 45–47] reported the effect of synoptic reporting on the overall completeness of a Pathology report (definition 1). Thirteen studies showed an increased overall completeness, independent of cancer type or synoptic reporting level of the module. In contrast, only one article [33] described that the SR was less complete than the NR

Out of the 14 studies [21, 23–25, 28, 30, 32, 33, 36, 38, 40, 45–47] that reported the effect of SR on the overall completeness of a pathology report, 13 showed an increased overall completeness, for several cancer types and SR levels (Fig. 2). SR was associated with an increased probability of providing information on the mandatory parameters [23–25] and a decrease in the number of missing parameters in a pathology report [36, 48]. The study that failed to show improved completeness [33] commented on the restricted list of parameters in the SR as defined by CAP. For example, in the guidelines as defined by CAP, SR description of specimen type lacked specific histological codes, whereas in NR, these histological codes could be included.

#### Parameter-specific completeness

Five studies described the impact of SR on parameter-specific completeness in breast cancer. Four studies described the implementation of SR level 3 (Table 2). The results of the fifth article of Branston et al. [28], which implemented SR level 4, were calculated as the percentage change in minimum dataset completeness; these data are excluded from the table. ‘Tumour type’ and ‘lymph node status’ were already reported sufficiently in NR. The ‘oestrogen receptor’ and ‘progesterone receptor’ were already reported sufficiently in NR according to two studies [19, 40], but for another study, implementation of SR was needed to achieve a sufficient reporting [41]. McEvoy et al. reported increased completeness of the oestrogen receptor from 84 to 99 %; however, a decrease was seen for the progesterone receptor [41]. The implementation of SR led to an increased completeness of four parameters (‘resection margins’, ‘DCIS size’, ‘location: quadrant’ and ‘calcification’). Three parameters increased significantly in the majority of the studies ‘histological grade’ [19, 23, 40, 41], ‘lymphovascular invasion’ [19, 23, 41] and ‘lesion size’ [19, 23] or already showed sufficient completeness in NR [19, 40]. The parameters ‘distance tumour to resection margin’, ‘type of specimen’, ‘location side’, ‘multiple tumour foci’ and ‘CIS in specimen’ showed diverse results; in some studies, the parameters were already sufficiently reported in NR, whilst in other studies, implementation of SR was necessary.

**Table 2:** Parameter-specific completeness of the breast cancer pathology report

Article	Appleton [23]		Austin [19]		Mathers^a^ [40]		McEvoy [41]		
Level SR	Level 3		Level 3		Level 3		Level 3		
Origin Guideline	NHSBSP		ACN		RCP		NHMRC		
Reporting format	NR	SR	NR	SR	NR	SR	NR	SR2	SR
Number of reports	30	10	95	307	50	50	385	584	680
Individual parameters (%)									
Lesion size	63.3	100*	98.9	100	80	88			
Tumour type	93.3	100	97.9	100	100	98	100	100	100
Histological grade	70	100*	86.3	100*	96	100	50.1	86.1	97.5*
Lymph node status	100	100	100	99.6	100	100	100	100	100
Resection margins	80	100	89.5	96.1*					93.2
Lymphovascular invasion	70	100	89.5	99.7*	98	98	31.2	66.8	96.9*
CIS in specimen	80	100*	95.8	98.1	84	100*	100	95.7	99.7
DCIS size	23.67	100*	43.9	65.9					
Type of specimen	46.67	100*	100	100					
Location, side	43.33	100*	100	100					
Location, quadrant	76.67	100*	30.5	46.6*					
Multiple tumour foci			22.1	74.3*			98.2	97.4	100
Calcification			36.8	91.9*	82	100			
ER status			94.5	94.1	90	100	83.9	67.6	98.7*
PR status			93.4	88.5	90	98	83.6	67.3	71.8*

*NHSBSP* National Health Service Breast Screening Programme, *ACN* Australian Cancer Networ, *RCP* Royal College of Pathologists, *NHMRC* National Health and Medical Research Council, *NR* narrative report, *SR* synoptic report; SR2 approximately 50 % reported synoptically, *ER* oestrogen receptor, *PR* progesterone receptor

*Significant improvement in completeness according to the article

^a^Mastectomy and biopsy merged together

**Fig. 3:**
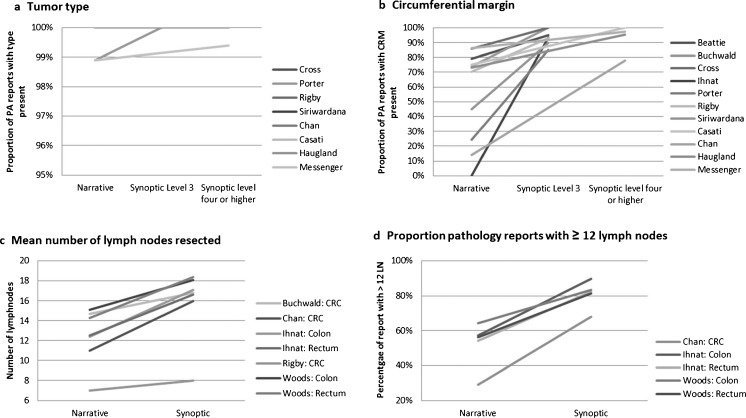
Impact of synoptic reporting on individual parameters in a colorectal specimen pathology report. **a** The effect of synoptic reporting on the proportion of pathology reports containing information on tumour type in colorectal cancers. **b** The effect of synoptic reporting on the proportion of pathology reports containing information on circumferential margin in rectal cancers. **c** The effect of synoptic reporting on the absolute mean number of lymph nodes resected per resection specimen. **d** The effect of synoptic reporting on the proportion of pathology reports reported 12 or more lymph nodes resected

Fourteen studies on SR of colorectal cancer described a quantitative effect on parameter-specific completeness. Of these 14 studies, 13 are represented in Table 3. For colorectal cancer, we merged colon and rectal cancer data if reported separately. The results of the 14th article by Branston et al. [28], which implemented SR level 4, were calculated as the percentage change in minimum dataset completeness. These were excluded from the table. Nine studies described the effect of implementing SR level 3, and five studies described the effect of implementing SR level 4 or higher. Four individual parameters were already reported sufficiently in NR (tumour type (Fig. 3a), ‘depth of invasion’, ‘total lymph nodes’ and ‘lymph nodes with metastasis’). ‘Tumour size’ was adequately reported in the NR of three studies [18, 42, 46] but lacking in a fourth [21]. ‘Histological grade’ was sufficiently reported in the majority of studies (*n* = 9) but not in three other studies [26, 28, 36]. The completeness of both parameters was increased to 96–100 % after the introduction of SR. The implementation of SR led to increased completeness for the reporting of the ‘circumferential resection margin’ (Fig. 3b), ‘distant resection margins’, ‘perineural invasion’ and ‘vascular and lymphovascular invasion’. The parameters ‘stage’, ‘resection margin’ and ‘nodal status’ showed diverse results; in some studies, NR was already very good, whilst in other studies, the implementation of SR was necessary.

**Table 3:** Completeness of the colorectal carcinoma pathology report per individual parameter

Article		Beattie [26]		Buchwald [29]		Cross [31]		Idowu [36]		Ihnat^b^ [37]		Porter [43]		Rigby [46]		Siriwardana [21]		Woods^b^ [51]		Chan [18]			Casati [30]		Haugland [34]			Messenger [42]	
Level SR		Level 3		Level 3		Level 3		Level 3		Level 3		Level 3		Level 3		Level 3		Level 3		Level 4			Level 5		Level 5			Level 6	
Guideline		RCP		Own		RCP		CAP		CAP		Own		RCP		RCP		RCP		CAP			RCP		RCP			CAP	
Localisation		CRC		CRC		CRC		CRC		CRC		Rectum		CRC		CRC		CRC		CRC			CRC		CRC			Rectum	
Reporting format		NR	SR	NR	SR	NR	SR	NR	SR	NR	SR	NR	SR	NR	SR	NR	SR	NR	SR	NR	SR	SR3	NR	SR	NR	SR4	SR5	NR	SR
Number of reports		85	86	97	205	43	68	414	665	84	93	177	20	54	44	82	68	549	404	108	116	113	123	1089	368	112	170	183	315
Individual parameter (%)																													
Tumour size														100	100	88	100*			100	100	100						98.8	99.4
Tumour type						100	100					98.9	100	100	100	100	100			100	100	100	100	100	100	100	100	98.9	99.4
Histological grade		86	100*			100	100	76.8	96*	100	100	94.4	100	100	100	98	100			98	100	100	98.4	99.9	96.7	100	99.4	91.8	97.5
Resection margins						54	100*	41.5	84*	100	100					91	100*			50	99*	100	80.5	99.7*	84	90.2	97.1*		
CRM		79	95	74	100*	86	100*			0	93.3*	24.3	85*	70.4	93.2*	45	90*			14	64*	78	75	100*	73.3	94.9	95.5*	86.3	97.4*
Invasion																													
	Vascular	68	100*	52	98*	88	100*			44	76.3			98.2	97.7	80	100*	30.6	40.3	18	88*	100						41	96.8*
	Perineural			52	98*					23.8	52.7									7	88*	100						13.7	94.0*
	Lymphovascular											55.9	90*							81	97*	100						39.3	98.1*
Stage^a^		39	100*			100	100			100	100	14.1	80*	98.2	100	77	100*						58.2	100*				24	96.2*
Depth invasion						100	100													100	100	100	100	100	96.7	100	100		
Ln status																85	99*	60.5	82.4*	100	100	100						99.8	100
Total LN												91	100										98.4	99.9	97.6	99.1	100		
LN with metastasis												91	100										99.2	99.7	97.6	99.1	100		

*RCP* Royal College of Pathologists, *CAP* College of American Pathologists, *CRC* colorectal carcinoma, *NR* narrative report, *SR* synoptic report, *SR3* synoptic reporting 15 months after implementation, *SR4* local synoptic report, *SR5* national synoptic report, *CRM* circumferential margin (only on rectum tumours)

*Significant improvement in completeness according to the article

^a^TNM, but in Beattie [26] and Cross [31], Dukes was used

^b^Colon and rectum are merged

Eight studies on SR described other cancer types, as shown in the tables (Online resources 2–6). Common parameters ‘tumour size’, ‘histological type’ and ‘histological grade’ were already reported sufficiently in NR, whereas for ‘resection margins’ and ‘(lympho)vascular/perineural invasion’, implementation of SR was necessary for an increased completeness to 96–100 %.

#### Quality of pathology evaluation

Implementation of SR is also expected to affect the quality of pathology evaluation. One aspect of quality is the accurate ascertainment of nodal tumour metastasis. If more lymph nodes are being resected, the N stage will be reported more accurately. For colorectal cancer, it is advised internationally to resect at least 12 lymph nodes [53]. The mean number of lymph nodes identified in the surgical specimen for colorectal cancer was evaluated in 5 of the 14 included studies [18, 29, 37, 46, 51]. All studies showed improvement in mean number of lymph nodes after implementation of SR (Fig. 3c), and more frequently, the minimum number of 12 lymph nodes was achieved. Three studies also showed an improvement of the proportion of pathology reports with a minimum of 12 lymph nodes reported after implementation of SR (Fig. 3d).

## Discussion

In this systematic review, we showed that SR results in more complete pathology reports. Whilst traditional parameters such as ‘tumour type’, ‘grade’, ‘invasion depth’ and ‘nodal status’ are in general well reported with NR, other clinical relevant features such as resection margins and ‘type of local spread (vascular, lymphovascular and perineural invasion)’ are frequently lacking. The introduction of SR results in improved reporting of these parameters. SR also improves the mean number of lymph nodes reported and the proportion of pathology reports with 12 or more lymph nodes [53].

Besides these favourable quantitative outcomes, pathologists found that SR was quick and easy to complete and that reports included all essential parameters [28]. Even though SR appears to be more time-consuming in the beginning, implementation actually resulted in a significant reduction time spent on the production of the report by pathologists [54, 55]. For multidisciplinary meetings, both pathologists and clinicians appreciated consistency of the reports [56]. Necessary information for patient management was quick and unambiguous to find [28, 56].

SR can be implemented in different ways. In the studies included in the present review, the following six different implementation strategies were described: combined implementation of SR with clinical audits [23, 31, 47], organisation of SR education or meetings [18, 28, 32, 37], attachment of SR hard copy to the request form of the resection specimen [21, 31, 44], addition of explanatory notes to the SR [20, 24], mandated inclusion of essential parameters according to guidelines [33, 36, 51] or introduction of the SR module without any special attention [19, 26, 29, 30, 38, 41, 42, 46]. The implementation strategy could partially explain the success of implementation of SR. Srigley et al. described the implementation of SR in Ontario, Canada, where pilots and audits were used to ensure proper implementation of SR. In 2012, they achieved successful implementation in 92 % of all hospitals in Ontario [6]. In addition, funding for hospitals, as was used in Ontario [6, 47], could also have added to the successful implementation of SR.

To date, SR has not been widely adopted in anatomic pathology reporting. The main barriers preventing successful implementation are the personal preference of pathologists, who like the flexibility and work flow of NR [57]. Whilst indeed initially, introduction is likely to disrupt the work flow, this seems a temporary situation. Flexibility is sometimes necessary to express uncertainty about a diagnosis; this can in most cases be solved by addition of free text fields to a SR. For instance, Hassel et al. [58] reported that pathologists found the SR more difficult and inflexible and they missed parameters. Another factor hampering implementation is the introduction of the new reporting format in existing work environments, such as the electronic patient files and software systems used throughout the hospital [57, 59]. As reported by Bjugn et al. [27], the development of the SR in Norway was delayed considerably because of alterations in the mandatory diagnostic criteria of the SR and because of alterations in the user interface for the SR.

There are some potential limitations to our study. We are confident that with our search, we found the majority of published papers, minimising the risk of selection bias. However, publication bias may cause an overrepresentation of positive study outcomes.

All studies in this review were observational. The design was either case-control or cross-sectional. No randomised controlled trial has been conducted on the effect of SR on pathology reporting. However, in our opinion, a retrospective study is suitable to investigate the effect of SR in practice. Eight studies reported the effect of SR in less than 200 reports [20, 21, 23, 37, 40, 43, 46, 48]; this is partly due to manually auditing the data for completeness. Preferably, future studies would include much higher number of pathology reports to get a better understanding of the impact of SR on pathology reporting. The fact that these studies cover different tumour types and are conducted in different countries and continents increases the generalisability. Even though most articles investigated the effect of breast and/or colon cancer, we expect that the results reported in this review are transferable to implementation of SR for other cancer types and countries not yet investigated.

Based on the current data, we can conclude that SR results in improved reporting of clinical relevant data. For this reason, it is our opinion that SR is already at present the best clinical practice for anatomic pathology cancer reporting. Ongoing innovation in SR software will likely further improve the value of SR in anatomic pathology, as well as improve the ease of use and efficiency of reporting with SR modules.

## Electronic supplementary material

Supplementary information is available at Virchows Archive website.

ESM 1 (DOCX 43 kb)
