# Supplementary material for: The effects of implementing synoptic pathology reporting in cancer diagnosis: a systematic review
Source: Virchows Arch. 2016 Apr 21;468:639–49. doi: 10.1007/s00428-016-1935-8 (PMC4887530; doi:10.1007/s00428-016-1935-8)
Supplement: Supplementary file 1 — (DOCX 43 kb) [file 428_2016_1935_MOESM1_ESM.docx]

# Supplementary material The effects of implementing synoptic pathology reporting in cancer diagnosis: A systematic review

Caro E. Sluijter^1,2^, Luc R.C.W. van Lonkhuijzen^3^, Henk-Jan van Slooten^2,4^, Iris D. Nagtegaal^1,2^, Lucy I.H. Overbeek^2^

1. Department of Pathology, Radboud University Medical Centre, Nijmegen, the Netherlands
2. Foundation PALGA (the nationwide network and registry of histo- and cytopathology in the Netherlands), Houten, the Netherlands
3. Centre for Gynaecological Oncology, Academic Medical Centre, Amsterdam, the Netherlands
4. Symbiant Pathology Expert Centre, Alkmaar, the Netherlands

Corresponding author:

Caro E. Sluijter, MSc

Radboud University Medical Centre,

Department of Pathology, Huispost 824

P.O. Box 9101, 6500 HB Nijmegen, the Netherlands

Email: [caro.e.sluijter@radboudumc.nl](mailto:caro.e.sluijter@radboudumc.nl)

| Supplementary Table 1: Search strategy for systematic review | | |
| --- | --- | --- |
| Search engine | Search strategy | Items found |
| Pubmed | #1: Search ((((checklist*[tw] OR template*[tw] OR synoptic[tw] OR proforma[tw] OR guideline*[tw] OR standard*[tw])))) AND ((Pathology report[tw] OR Pathologic report[tw] OR Histopathology report[tw] OR Histopathologic report[tw] OR histo-pathology report[tw] OR histo-pathologic report[tw] OR Pathology reports[tw] OR Pathologic reports[tw] OR Histopathology reports[tw] OR Histopathologic reports[tw] OR histo-pathology reports[tw] OR histo-pathologic reports[tw] OR Pathology reporting[tw] OR Pathologic reporting[tw] OR Histopatholgy reporting[tw] OR Histopathologic reporting[tw] OR histo-pathology reporting[tw] OR histo-pathologic reporting[tw])) | 975 |
|  | #2: Search (((report[tw] OR reporting[tw] OR reports[tw]))) AND (("Pathology, Clinical/standards"[Mesh]) OR "Pathology, Surgical/standards"[Mesh]) | 425 |
|  | #3: Search #1 OR #2 | 1272 |
|  | #4: Repeat search #3 at 01/01/2015 | 66 |
| Embase | ((checklist* or template* or synoptic or proforma or guideline* or standard*) and (Pathology report or Pathologic report or Histopathology report or Histopathologic report or histo-pathology report or histo-pathologic report or Pathology reports or Pathologic reports or Histopathology reports or Histopathologic reports or histo-pathology reports or histo-pathologic reports or Pathology reporting or Pathologic reporting or Histopathology reporting or Histopathologic reporting or histo-pathology reporting or histo-pathologic reporting)).mp. | 1580 |
| Cochrane | ((checklist* OR template* OR synoptic OR proforma OR guideline* OR standard*) AND (Pathology report OR Pathologic report OR Histopathology report OR Histopathologic report OR histo-pathology report OR histo-pathologic report OR Pathology reports OR Pathologic reports OR Histopathology reports OR Histopathologic reports OR histo-pathology reports OR histo-pathologic reports OR Pathology reporting OR Pathologic reporting OR Histopathology reporting OR Histopathologic reporting OR histo-pathology reporting OR histo-pathologic reporting)) | 244 |
| Total |  | 3162 |

| Supplementary Table 2: Parameter specific completeness of the pancreas cancer pathology report | | | | | | | |
| --- | --- | --- | --- | --- | --- | --- | --- |
| Article | Gill [21] | | Westgaard [49] | | | Westgaard [50] | |
| Level SR | Level 3 | | Level 3 | | | Level 3 | |
| Origin Guideline^a^ | CAP | | Expert opinion | | | Expert opinion | |
| Reporting format^b^ | NR | SR | NR3 | NR4 | SR | NR | SR |
| Number of subjects | 109 | 68 | 172 | 221 | 113 | 100 | 118 |
| Individual parameters (%) |  |  |  |  |  |  |  |
| Tumor size | 95 | 100 |  |  |  |  |  |
| Histological type |  |  | 31 | 21 | 88 |  |  |
| Histological Grade | 99 | 100 | 88 | 90 | 92 |  |  |
| Margins reported | 1 | 100* | 16 | 8,1 | 96* | 24.0 | 37.3 |
| Vascular invasion | 66 | 100* | 35 | 12 | 98* |  |  |
| Perineural invasion | 84 | 100* | 19 | 14 | 99* | 42.0 | 55.9 |
| Tumor infiltration |  |  | 74 | 70 | 99 |  |  |
| TNM stage | 56 | 100* | 31 | 40 | 100 |  |  |
| Nodal involvement |  |  | 42 | 50 | 60 | 31.0 | 57.6 |
| Poor differentiation |  |  | 31 | 29 | 30 | 19.0 | 38.1 |
| Pancreatic Tumor origin |  |  | 66 | 72 | 46 |  |  |
| Cancer origin discussed |  |  | 85 | 80 | 97 |  |  |
| Premalignant changes |  |  | 15 | 11 | 48 |  |  |
| Estimation Tumor size |  |  | 56 | 41 | 95 |  |  |
| a: CAP = College of American Pathologists  b: NR = Narrative report; SR = Synoptic report; NR 3 = narrative reporting low volume hospital; NR 4 = narrative reporting medium volume hospital  * = significant improvement in completeness according to the article | | | | | | | |

| Supplementary Table 3: Parameter specific completeness of the melanoma pathology report | | | | | | | |
| --- | --- | --- | --- | --- | --- | --- | --- |
| Article | Haydu [35] | | | Karim [39] | | | |
| Cancer | Melanoma | | | Melanoma | | | |
| Origin Guideline^a^ | AJCC/UICC | | | AJCC/UICC | | | |
|  |  |  |  | SMU reports^c^ | | Non-SMU reports | |
| Reporting format^b^ | NR | SR | Combined | NR | SR | NR | SR |
| Number of subjects | 554 | 410 | 433 | 184 | 671 | 604 | 233 |
| Individual parameter (%) | | | | | | | |
| Breslow thickness | 99.1 | 100 | 100 | 97.3 | 100 | 91.1 | 99.6 |
| Level of invasion (Clark) | 96.2 | 99.2 | 100 | 90.2 | 100 | 83.6 | 100* |
| Dermal mitotic index | 78.9 | 98.8*^b^ | 98.8 | 79.3 | 100* | 78.1 | 99.6* |
| Ulceration | 75.6 | 98.5* | 99.3 | 67.4 | 99.6* | 53.5 | 99.6* |
| Invasive peripheral margin | 28.9 | 68.0* | 83.4* | 60.9 | 99.7* | 54.4 | 98.7* |
| Deep margin | 45.1 | 94.4* | 95.6 | 47.8 | 99.7* | 38.7 | 94.8* |
| Histopathological subtype | 63.2 | 98.5* | 97.2 | 57.6 | 99.7* | 64.6 | 96.1* |
| Vascular invasion | 74.2 | 98.8* | 99.5 | 62.0 | 99.9* | 53.6 | 97.0* |
| Lymphatic invasion |  |  |  | 25.0 | 99.9* | 19.4 | 54.1* |
| Neurotropism | 62.8 | 95.9* | 66.1 | 61.4 | 100* | 37.3 | 91.8* |
| Desmoplasia | 3.1 | 88.5* | 33.9 | 10.9 | 99.7* | 3.6 | 47.2* |
| Satellites | 6.1 | 79.8* | 91.9* | 12.5 | 100* | 74.7 | 5.1 |
| Regression | 59.6 | 99.5* | 99.5 | 60.9 | 100* | 39.9 | 97.4* |
| Predominant cell type | 38.8 | 93.9* | 54.3 | 41.8 | 99.6* | 35.6 | 67.8* |
| Associated naevus | 36.6 | 91.2* | 64.5 | 37.5 | 99.4* | 28.3 | 82.8* |
| a: AJCC/UICC: American Joint Committee on Cancer/Union for International Cancer Control  b: NR = Narrative report; SR = Synoptic report; Combined = combination of synoptic report and narrative report  c: SMU = Sydney melanoma unit (specialized); non-SMU = other non-specialized melanoma medical unit  * = significant improvement in completeness according to the article | | | | | | | |

| Supplementary Table 4: Parameter specific completeness of the prostate cancer pathology report | | | | | |
| --- | --- | --- | --- | --- | --- |
| Article | Aumann [24] | | | Ventura [48] | |
| Level SR | level 5 | | | Level 3 | |
| Origin Guideline^a^ | CAP | | | CAP | |
| Reporting format^b^ | NR | STR | SR | NR | SR |
| Number of subjects | 411 | 333 | 305 | 83 | 83 |
| Individual parameters (%) |  |  |  |  |  |
| Histological tumor type | 92.9 | 99.7* | 99.7 |  |  |
| Margins reported | 81.3 | 97.1 | 99.7* |  |  |
| Vascular invasion | 21.4 | 83.5* | 100* |  |  |
| Perineural invasion | 33.8 | 85.0* | 100* | 30.1 | 100 |
| Lymphovascular invasion | 24.8 | 83.5* | 100* | 27.7 | 100 |
| TNM stage | 85.3 | 97.9 | 99.7 | 43.4 | 100 |
| Lymph node status | 99.5 | 100 | 100 |  |  |
| Intra-prostatic tumor spread | 75.2 | 85.0* | 99.3* |  |  |
| Extra-prostatic extension | 25.6 | 70.3* | 98.3* |  |  |
| Seminal vesicle involvement | 79.8 | 88.6* | 100* |  |  |
| Gleason score | 98.9 | 99.4 | 100 |  |  |
| a: CAP = College of American Pathologists  b: NR = Narrative report; STR = Structured/checklist; SR = Synoptic report  * = significant improvement in completeness according to the article | | | | | |

| Supplementary Table 5: Parameter specific completeness of the lung cancer pathology report | | | |
| --- | --- | --- | --- |
| Article | Aumann [25] | | |
| Level SR | level 5 | | |
| Origin Guideline | Expert opinion | | |
| Subject organ | Lung | | |
| Reporting format^a^ | NR | STR | SR |
| Number of subjects | 246 | 415 | 214 |
| Individual parameters (%) |  |  |  |
| Tumor size | 80.7 | 97.3* | 100 |
| Tumor location | 69.9 | 51.1 | 100* |
| Histological type | 87.1 | 97.8* | 100 |
| Histological Grade | 97.6 | 99.5 | 100 |
| Margins reported | 96.2 | 99.7 | 100 |
| Vascular invasion | 24.9 | 82.9* | 96.7* |
| TNM stage | 99.6 | 99.5 | 100 |
| Lymph node status | 100 | 99.8 | 100 |
| Involvement of the pleura visceralis | 49.4 | 75.7* | 99.1* |
| Atelectasis / obstructive pneumonitis | 51.4 | 70.1* | 84.6* |
| a: NR = Narrative report; STR = Structured/checklist; SR = Synoptic report  * = significant improvement in completeness according to the article | | | |

| Supplementary Table 6: Parameter specific completeness of the uterine/cervix cancer pathology report | | | |
| --- | --- | --- | --- |
| Article | Reid [44] | | |
| Level SR | level 3 | | |
| Origin Guideline | Expert opinion | | |
| Reporting format^a^ | NR | NR2 | SR |
| Number of subjects | 115 | 36 | 198 |
| Individual parameters (%) | | | |
| Epithelium present | 6 | 98 | 14 |
| Transformation zone | 39 | 0 | 22 |
| CIN presence | 100 | 100 | 100 |
| CIN grade | 100 | 100 | 100 |
| Growth, expansile | 4 | 15 | 2.8 |
| CGIN, invasive | 4 | 1.5 | 8.3 |
| Number transactions involved | 72 | 99.5 | 81 |
| Location CIN | 65 | 99 | 64 |
| Excision margin, endocervical | 50 | 98 | 64 |
| Excision margin, ectocervical | 22 | 97 | 47 |
| Both excision margins | 45 | 1 | 31 |
| HPV changes | 35 | 98 | 42 |
| HPV changes positive | 35 | 81 | 42 |
| HPV changes negative | 0 | 16 | 0 |
| Glandular epithelium status | 25 | 99 | 50 |
| Inflammation | 10 | 93 | 19 |
| Summary line | 88 | 100 | 75 |
| Smear correlation | 5.2 | 95 | 8.3 |
| a: NR = Narrative report report before implementation of synoptic reporting; NR 2 = narrative report after implementation of synoptic reporting; SR = Synoptic report  * = significant improvement in completeness according to the article | | | |
